# Alkaloids Used as Medicines: Structural Phytochemistry Meets Biodiversity—An Update and Forward Look

**DOI:** 10.3390/molecules26071836

**Published:** 2021-03-25

**Authors:** Michael Heinrich, Jeffrey Mah, Vafa Amirkia

**Affiliations:** 1 Research Group ‘Pharmacognosy and Phytotherapy’, UCL School of Pharmacy, University of London, 29–39 Brunswick Sq., London WC1N 1AX, UK; jeffrey.mah.18@ucl.ac.uk (J.M.); v.amirkia.12@alumni.ucl.ac.uk (V.A.); 2 Graduate Institute of Integrated Medicine, College of Chinese Medicine, and Chinese Medicine Research Center, China Medical University, No. 100, Section 1, Jingmao Road, Beitun District, Taichung 406040, Taiwan

**Keywords:** alkaloid development, natural products, drug-likeness ethnopharmacology, drug discovery, biodiversity, GBIF (Global Biodiversity Information Facility), modelling

## Abstract

Selecting candidates for drug developments using computational design and empirical rules has resulted in a broad discussion about their success. In a previous study, we had shown that a species’ abundance [as expressed by the GBIF (Global Biodiversity Information Facility)] dataset is a core determinant for the development of a natural product into a medicine. Our overarching aim is to understand the unique requirements for natural product-based drug development. Web of Science was queried for research on alkaloids in combination with plant systematics/taxonomy. All alkaloids containing species demonstrated an average increase of 8.66 in GBIF occurrences between 2014 and 2020. Medicinal Species with alkaloids show higher abundance compared to non-medicinal alkaloids, often linked also to cultivation. Alkaloids with high biodiversity are often simple alkaloids found in multiple species with the presence of ’driver species‘ and are more likely to be included in early-stage drug development compared to ‘rare’ alkaloids. Similarly, the success of an alkaloid containing species as a food supplement (‘botanical’) is linked to its abundance. GBIF is a useful tool for assessing the druggability of a compound from a certain source species. The success of any development programme from natural sources must take sustainable sourcing into account right from the start.

## 1. Introduction

Drug discovery strategies have changed considerably over the last decades. Selecting candidates for drug developments using computational design and empirical rules has resulted in a broad discussion about their success [1]. “Maximal chemical diversity” and “druggability” questions, for example, are tackled through the works of Lipinski [2] which involves log P, molecular weight and hydrogen bond acceptors and donors to predict pharmacokinetic properties for lead compounds. Other metrics for lead selection include ligand efficiency [3], rotatable bond [4], and polar surface area [4] for absorption predictions. Screening libraries used in drug discovery are anything but diverse, since the rules of chemical synthesis filter out many diversified promising lead compounds. 

Even though natural products have been a source of medicine dating back to at least 2600 BC [5] with a huge impact on modern medicine discovery [6], many of the current empirical rules and filters lack the considerations for molecules with diversified properties [2], especially in natural products like alkaloids basic, cyclic organic compound containing nitrogen in the ring systems. While many alkaloids are classified according to their molecular skeletons, classification based on botanical origins are also used [7]. Alkaloids provided unique lead compounds for medicine. They have basic properties, in which they are water soluble under acidic conditions and lipid soluble under neural and basic conditions. This is especially important for dissolution in protonated form and membrane permeation in deprotonated form. 

Alkaloids are mainly biosynthetically derived from amino acids resulting in variety of chemical structures, mostly isolated from plants [7]. Alkaloids can be found in about 20% of plant species in small qualities [8] and their production (including in biotechnology), extraction and processing remain major areas of research and development [9,10]. Alkaloid biosynthetic pathways can be manipulated genetically for example in order to achieve higher production levels of alkaloids [11].

There is a need for drug discoveries from natural sources to result in a more diversified medicine portfolio for human use. Furthermore, natural products are more likely to resemble endogenous metabolites and biosynthetic intermediates compared to synthetic compounds which can be recognized as substrate by active transporters [12]. Despite the changes in discovery strategies and most notably the emergence of medicines derived from molecular biology, there remains a need to develop natural product-based medicines which has shown great success as a strategy.

Alkaloids play an essential role in both human medicine and in an organism’s natural defence. Alkaloids make up approximately 20% of the known secondary metabolites founds in plants [13]. In plants, alkaloids protect plants from predators and regulate their growth [14]. Therapeutically, alkaloids are particularly well known as anaesthetics, cardioprotective, and anti-inflammatory agents. Well-known alkaloids used in clinical settings include morphine, strychnine, quinine, ephedrine, and nicotine [15]. Recently, there is a resurgence of interest in bioactive natural products, driven both by a very proactive development in the field of traditional medicines (ethnopharmacology) as well as their potential in drug discovery [16]. As of 25 October 2020, 27,683 alkaloids were included in the Dictionary of Natural Products (DNP) with 990 hits of newly reported or reinvestigated alkaloids from nature between 2014 to 2020. 

The GBIF database has been used extensively in environmental research and is based on gathering species occurrence to assess the global distribution of each. GBIF occurrences are documented using preserved and living specimens, along with other ecological observations that include geographical data and the date of the record. These include collected and documented specimens, citations, and records of species including cultivated and wild occurences. In 2014, the GBIF database contained 424,254,844 occurrences of organisms in nature including 117,909,945 (27.8%) records from the kingdom Plantae. Despite nearly half a billion species having been recorded, many species demonstrated zero occurrences due to inadequate data reporting such as the non-participating countries for GBIF contributions and species inaccessibility.

Important strategic changes have been incorporated for data quality and data abundance. The GBIF database is developed using information from organizations (institutions, networks, and societies) and citizen scientists through participating projects that publish datasets through the GBIF network. Many countries remain unconnected with GBIF, which therefore aims to establish connections with these countries, thereby improving national capacity to access and mobilize biodiversity data among existing participants [17]. Compared to 76 participating countries in 2014, GBIF had over 100 participants in 2020 including countries (national government), economies, and international organizations contributing to GBIF data. It also aims to identify gaps and bias in data to achieve the highest quality data possible. One example is the updated data validator, which can flag duplicate records, inconsistences in formatting, and incomplete fields of necessary information [17]. 

In 2014, Amirkia and Heinrich [18] showed that historically, pharmaceutical alkaloids’ market performance has been linked to the abundance of a species as defined by the GBIF dataset, which is the most comprehensive database defining the distribution of individual species currently available. However, alkaloids are underrepresented as lead compounds to discover new medicines’ marketing and licensing [19]. This analysis was based on 117,909,945 records from the kingdom Plantae (27.8% of all organisms’ total entries). Since 2014, GBIF has added 204,963,126 records, a 3.82-fold increase. The larger sample size for species distribution from the previous sample size in 2014 gathered by Heinrich and Amirkia (2014) [18] enables a more accurate and precise analysis of global alkaloid abundances and biodiversity. Therefore, this paper aims to *assess the relevance of a species’ geographical abundance for a compound’s ‘druggability’ using a larger dataset and expanding this perspective to alkaloid containing plants used as supplements (botanicals).*

## 2. Results and Discussion

### 2.1. GBIF Data to Assess Species That Contain Alkaloid Abundances around the Globe

With growing interest in biodiversity, occurrences in GBIF for all kingdoms have grown 3.77-fold. As of 2020, the GBIF database contains 1,619,239,460 occurrences of organisms in nature including 322,873,071 (20%) records from the kingdom Plantae with a 2.73-fold increase for occurrences of ‘Plantae’ and a 3.82-fold increase of all records. With a larger sample of data and diversified species, assessments such as species richness patterns are resilient to the problematic occurrences’ records. However, there are a few species such as *Lycopodium complanatum* L., *Cocos nucifera* L., and *Lachnanthes tinctoria* (Lam.) Dandy with decreased occurrences in 2020 datasets. This is presumably linked to the removal of replicates from GBIF [20] or data lost through attempts to filter it [21], resulting in a rapid increase in its availability.

The GBIF database reported an 8.86-fold increase in occurrences (Table 1) of species containing alkaloids in 2020 compared to the data gathered by Amirkia and Heinrich in 2014 [18]. This is substantially higher than the overall rise for the kingdom Plantae, demonstrating only a 3.82-fold increase. This substantial difference signifies the importance of alkaloids in plant cultivation and natural biodiversity. The standard deviation in 2020 data is significantly higher (8.33-fold greater) than the data in 2014 (Table 1) despite having the same number of identified alkaloids. A t-paired test (t 0.99) was performed, confirming the significant increase for both standard deviations and averages in alkaloids containing species. The rapid growth in alkaloids containing species recorded suggests a global interest in exploring their potential. The higher standard deviations imply a wider variation of alkaloid producing natural sources around the globe. Furthermore, the increased standard deviation points to differences in recording strategies of biodiversity, signifying that the successful development of medicinal products continues to rely on adequate biodiversity to meet supplies for market demands [18].

### 2.2. Marketed Alkaloids and Source Plants—An Update Based on GBIF2020

From analysing 24,325 alkaloids after filtering initial sets of 27,683 gathered from the ‘Dictionary of Natural Products’ web portal, only 0.002% (52/27,683) of these alkaloids were used as licensed medicines. According to the GBIF dataset (2020), species that yield medicinal alkaloid occurrences have a 3.31-fold higher level of occurrence compared to GBIF dataset in 2014. Furthermore, medicinal alkaloids such as Galanthamine and Yohimbine can be derived from different genera without being listed under specified species which may result in an underestimation. Therefore, the GBIF occurrences of medicinal alkaloids are the minimum based on known records at a species level from the Dictionary of Natural Products. Nevertheless, despite the relatively small increase, medicinal alkaloids continue to be highly abundant and explored compared to non-medicinal alkaloids.

Alkaloids that have already been marketed (Table 2) were initially reported in Amirkia and Heinrich (2014) [18] and there have been no new plant-derived alkaloids that were licensed since 2014 to the best knowledge of the authors. However, there is a previously missed medicinal alkaloid that has been added to Table 2. In 2012, the pharmacologically active form of Omacetaxine mepesuccinate, derived from *Cephalotaxus harringtonia* (Knight ex J. Forbes) K. Koch [22] (syn.: *Cephalotaxus fortunei var. foemina* Carrière), was FDA-approved for chronic myeloid leukaemia (CML). This has paved the way for 57 alkaloids being identified for therapeutic use at time of writing, compared to the 56 identified in 2014.

There have been changes to the therapeutic uses of some of these alkaloids since initial market authorization. For example, ephedrine hydrochloride tablets were first given marketing authorization in 2007 for treatment or prevention of bronchospasm attacks in asthma. Later, ephedrine can also be used for neuropathic oedema although it is not licensed but indicated in the British National Formularies (BNF). Emerging clinical trials are investigating therapeutic potential in other diseases such as labour pain, vasopressors, and preeclampsia [23,24,25]. Another example is atropine, which in 2001 had licenses with the Medicines and Healthcare products Regulatory Agency (MHRA) for treatment like bradycardia and prevention of cholinergic effects on the heart after surgery. Further indicated uses now include eye diseases such as uveitis and cycloplegia. Clinical trials are also investigating other applications such as myopia and cataract treatment [26]. 

In 2014 Amirkia and Heinrich [18] highlighted that chondocurine and vincamine demonstrated less than ten occurrences in GBIF. In this updated analysis no alkaloid compound has less than ten occurrences, and all medicinal alkaloids (with the exception of Chondocurine) have more than fifty. 

Vincamine’s low incidence in 2014 was due to chemical and botanical nomenclature ambiguity. The ‘Dictionary of Natural Products’ (DNP) indicated “*Tabernaemontana rigida*” as a natural source for (+/−) isomers of Vincamine. After taxonomic validation in Medicinal Plant Names Services (MPNS), the accepted nomenclature for “*Tabernaemontana rigida*” is *Tabernaemontana muricata* Link ex Roem. and Schult. This nomenclature ambiguity significantly affected on the perception of its biodiversity. “*Tabernaemontana rigida*” has seven occurrences in the GBIF, while *Tabernaemontana muricata* Link ex Roem. and Schult reported 237 occurrences. Even more importantly, only (+)-vincamine is found naturally. Its stereochemistry has been produced by stereo/enantio-selective total syntheses, and absolute configuration through chiroptic methods (e.g., ORD) [27]. Due to the molecular rigidity of the (+) isomer, isomerization is unlikely to be due to stereo-specific biochemistry of plant enzymes in secondary metabolite synthesis [28]. This had not been represented in the DNP. With the inclusion of species such as *Vinca minor* L. that are well known as sources of therapeutic vincamine (identified as (+) isomer), the GBIF biodiversity jumped to approximately one hundred thousand occurrences. 

Medicinal plants are known to be particularly ‘weedy’ [29]. Often alkaloids are toxic and highly biologically active compounds that allow plants to rapidly colonize an area, especially in disturbed environments such as roadsides [30]. Clearly, species in accessible locations such as disturbed habitats or cultivated land are more likely to be harnessed. The accessibility and adaptive growth provided by alkaloid compounds in species enables exploration of other therapeutic uses. 

Many of the compounds listed above are now obtained from cultivated material and registered with the GBIF (Figure S1). Cultivated species and the many cultivars and varieties involved pose a particular problem [31]. Analysis of the species in Table 1 demonstrates many are now commonly grown or managed in order to secure the *materia prima*, and it is still problematic to assess such species within GBIF. Cultivated species are generally conserved ex situ in gene banks as well as conserved in active farming, and can be retrieved through a variety of databases such as Genesys, the European Search Catalogue for Plant Genetic Resources, Germplasm Resources Information Network, International Centre for Tropical Agriculture, Food and Agriculture Organization, and other national/regional gene banks [31]. Furthermore, only fractions of the vast databank for species information digitally available are without restrictions. Nonetheless, greater accessibility for medicinal alkaloid containing species drives research and development further, including opportunities to explore other therapeutic indications and potential uses (Table 2). 

### 2.3. Nonmedicinal Alkaloids and the Exploration of Future Medicinal Potential of Alkaloids

Analysing the yearly trend, based on the Web of Science Core Collection research output on the therapeutic potential of alkaloids increased steadily throughout the period of 2014 to 2020. The main research focus of alkaloids containing species is on pharmacology/pharmacy (17.53%), medicinal chemistry (9.96%), and plant sciences (13.24%). 

Seven plant species yielding non-medicinal alkaloids surpass one million occurrences (Table 3), although several of these are commonly under preclinical investigation in the context of drug development. The average occurrence of all alkaloid containing species is 11,210, with these seven occurring a hundred times more than average. Most of these alkaloids are found in multiple species, even across different families. For example, among the non-medicinal alkaloids, the pyrrolidine alkaloid 4-hydroxy-1,1-dimethylpyrrolidinium-2-carboxylate ((2R,4S)-4-hydroxy-1,1-dimethylpyrrolidinium-2-carboxylate; CID: 6604261), is linked to 1.3 million occurrences of its source species. These include *Achillea millefolium* L.*,* and Lamiaceae such as *Betonica officinalis* L., *Marrubium vulgare* L., and *Stachys sylvatica* L. Another example is the tropane alkaloid calystegine b [8-Azabicyclo [3.2.1] octane-1,2,3,4-tetrol; PubChem CID 124434] which can be extracted from a variety of species in the Solanaceae, including *Atropa belladonna* L.*, Solanum dulcamara* L., *Solanum tuberosum* L., *Datura wrightii* Regel. and related Convolvulaceae such as *Convolvulus arvensis* L. (syn. *Calystegia arvensis* L.). 

There has been a strong focus on investigating simple alkaloids as templates for drug discovery. The alkaloids indicated above are simple alkaloids (Table 3) in terms of the chemical structure. These alkaloids are abundant and thus potentially more easily extracted and readily available for research. Simple alkaloids are more abundant in nature due to their chemical simplicity and non-demanding (bio)synthesis pathways.

The abundance of these alkaloids prompts further investigation in preclinical trials. 4-hydroxy-1,1-dimethylpyrrolidinium-2-carboxylate (CID: 6604261) is often included in quantitative high throughput screening (qHTS) studies as indicated by the 111 bioassay-based studies that have been reported. However, most results are inconclusive or do not result in further research and development [32]. Although no conclusive results for its therapeutic uses have been reported, the common incorporation of 4-Hydroxy-1,1-dimethylpyrrolidinium-2-carboxylate for lead molecule identification reinforces the importance of abundance and accessibility in drug development.

Azabicyclo [3.2.1] octane-1,2,3,4-tetrol (CID: 124434) is currently being investigated pharmacologically for a wide spectrum of potential pharmacological effects, such as bacterial and human glucosylceramidase beta (GBA) inhibition [32]. GBA plays a major role in Gaucher disease as it is a lysosomal storage disorder caused by β-glucocerebrosidase activity deficiency. As of end of 2020, there are 8 bioassay-based studies (enzyme inhibition assays) that are active in ongoing investigations. [33,34].

Importantly for these alkaloids there is a ‘driver’ species (i.e., one that accounts for most of the biodiversity of alkaloid-bearing species). In the case of achillein (4-Hydroxy-1,1-dimethylpyrrolidinium-2-carboxylate), *Achillea millefolium* (990,000 occurrences) accounts for the majority of biodiversity linked to this compound. *A. millefolium* with a very wide distribution, contains less than ten identified simple and widely distributed alkaloids and is certainly not a classical source of alkaloid-containing drugs. *S. dulcamara* (363,000 occurrences) accounts for the majority of the biodiversity for Calystegine B (8-Azabicyclo [3.2.1] octane-1,2,3,4-tetrol). Both of the alkaloids are bioactive and reported to be the main chemical constituents in their respective species [35,36]. These two examples highlight that whilst abundance is a factor facilitating use as a medicine and scientific investigation, but clearly it is only one of several factors.

To quantitatively compare whether species abundance has an effect on research interests for simple alkaloids (focusing on those with a low molecular weight (less than 350 g/mol) and structurally simple (hydrogen bond acceptor less than 5, hydrogen bond donor less than 5, and PubChem complexity score less than 550) we identified alkaloids found in locally resticted species. These alkaloids are normally confined to one or two species in the same genus (Table 4). As expected, only very limited research outputs have been published. Most examples with a low GBIF distribution have been associated with between zero and four publications in Web of Science (Table 4), signifying the under-investigation of these alkaloids. These species are often endemic to specific regions in Africa, Oceania, or South America which creates a barrier for accessing these species.

*Bruguiera sexangula* and *Croton tiglium*, have about 40 research papers published with moderate to high occurrences for rare species. They are native to South Eastern and Southern Asian countries such as Malaysia, Vietnam, and Thailand. C. tiglium is a well-known medicinal and toxic species [37] found in local/traditional medicine preparations. It is also a source of co-carcinogenic phorbolesters, being one of the core species in early investigations of the phorbolesters’ pharmacological profile [38]. In traditional Chinese medicine it is used extensively for gastrointestinal disorders, intestinal inflammation, rheumatism, headache, peptic ulcer, and visceral pain [39]. In Chinese traditional medicine the mangrove shrub/tree *B. sexangula* is used mainly as for diarrhoea and detoxification [40]. Interest in this species seems to be linked to its unique habitat and the presence of endophytic fungi. Future alkaloid investigations are expected to increase research focus on phytochemical profiles.

For alkaloids in rare species with identified therapeutic activities, the focus of research shifts early on to the synthesis of the compound. *Stemona tuberosa* has been used in Korean and Chinese medicine for lung disease with reports of antifungal and antibacterial effects [41]. The active alkaloid Croomine exerts antitussive activity and exhibits a dose-dependent inhibition of coughing in a citric acid-induced guinea pig cough model [42]. Croomine attracted 40 research papers with 20 articles related to synthesis to counteract accessibility issues due to low chemo-diversity.

Another example *is M. tortuosum* with a highly restricted distribution (288 occurrences) in Africa. It has been used traditionally by Bushmen of Namaqualand as a stimulant and was also chewed frequently to quench thirst, suggesting its potential pharmacological intervention as a thirst or hunger suppressant [43]. Alkaloids in *M.* *tortuosum* have been reported as active secondary metabolites with a potential for a wider therapeutic use [44]. Mesembrine exerts great inhibitory potency for 5-HT transporter and PDE4 inhibition to treat anxiety and inflammatory disease [45] with non-toxic agents [46]. Pharmacological activities of Mesembrine attracted 163 research articles, with 90 including complete or partial synthesis of Mesembrine.

Simple alkaloids from species with low abundance did not attract high levels of research interest despite relatively simple extraction and identification requirements, as indicated by a low number of published research papers. Furthermore, these species (Table 4) are rarely used in traditional herbal medicine. Such uses therefore play a pivotal role in the development of inaccessible species, especially with regard to studying potential therapeutic benefits and risks. Both geographical locations and abundance play an essential role in drug discovery and this analysis provides an empirical basis for this theory. If species have extensive traditional use in medicines often associated with high local abundance, this may also trigger research activities.

### 2.4. Shifting Interests in Drug Discovery and Supplement Development

**Figure 1:**
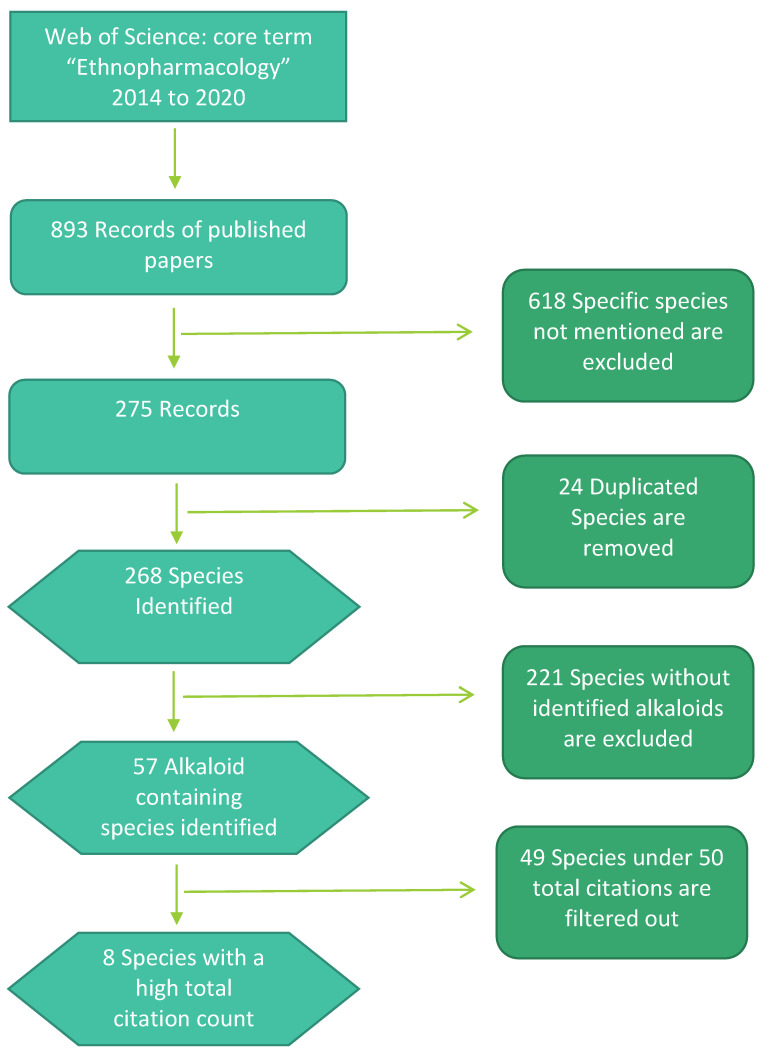
Flow chart indicating the selection strategy for identifying widely distributed alkaloid containing species currently researched as food supplements/botanicals

The above discussion emphasises the importance of species abundance in the development of new medicines. Similarly, questions can be asked regarding the research on medical preparations derived from medicinal plants, i.e., herbal medicinal products or ‘botanicals’. Therefore, in the next step we analysed the biodiversity distribution patterns of alkaloid containing species currently researched as food supplements/botanicals (Figure 1). We focused on clinical trials or intervention studies in the case of food supplements (Table 5), assessing whether bringing new medicinal and health food products onto the market is linked to a species’ abundance. These are selected based on total citations under the overarching term ethnopharmacology queried in Web of Science core collection between 2014 and 2020. DNP and CAS are cross referenced with the species to ensure the species contain alkaloids as secondary metabolites. All these alkaloid containing species have been used in traditional medicine, mainly in Traditional Chinese Medicine (TCM). Most clinical trials investigated possible uses of the whole botanical drug or its extract (as an herbal medicine, dietary supplements, etc.). Interestingly, in this case all species with the highest number of published studies are cultivated ones.

**Figure 2:**
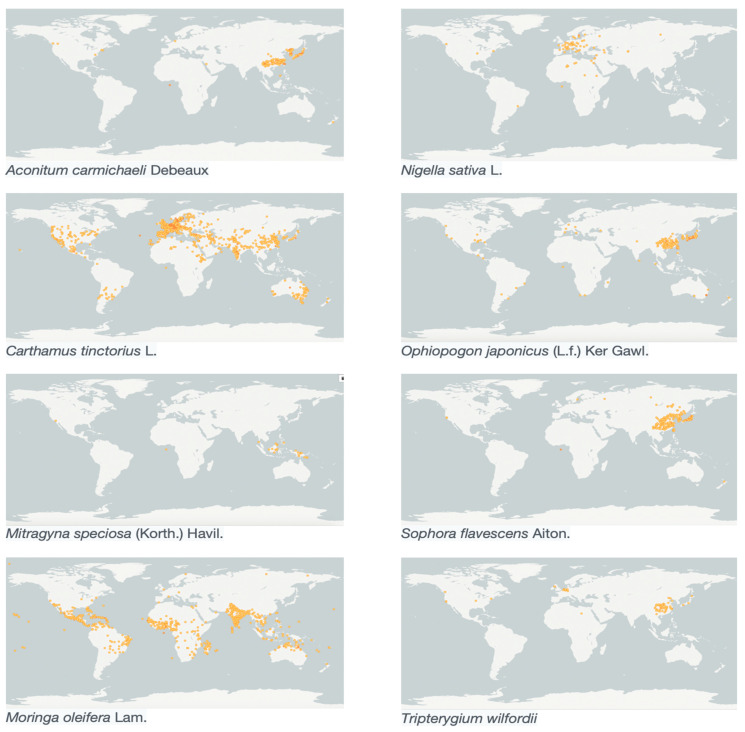
Geographical distribution of species used as supplements/botanicals containing alkaloids as bioactive substances. The intensity of orange areas demonstrates the level of abundances in the indicated area.

In terms of published papers (as indicators of research and development activities), as of 2020 there has been a notable increase for *Moringa olifera* and *Nigella sativa* (to 2931 and 2714 published papers, respectively), but not for the other botanical drugs analysed here. *M. oleifera* has been introduced mainly from India and Pakistan and cultivated intensively, found now as a cultigen around entire subtropical and tropical belts as well as in China and the United States [47] (Figure 2). It is fast-growing and drought-resistant, making it suitable for cultivation under ecologically difficult conditions. At the same time, multiple usage and reliability has attracted cultivation worldwide, especially in countries relying economically on the primary sector. Between the years of 2014 and 2020 *M. oleifera* demonstrated a significant increase in occurrences globally. Alkaloids in *M. oleifera* leaves, specifically for thiocarbamate glycosides, demonstrated clinical importance with reported antimicrobial [48], antitumour [49], and antihypertensive [50] properties. In recent clinical trials, *M. oleifera* (leaves) have been investigated as treatments for type 2 diabetes mellitus, metabolic syndrome, osteoporosis, dyslipidaemias, HIV infections, malnourishment, and postpartum Disorders. It also demonstrated cardioprotective properties linked to thiocarbamate alkaloids which explicated protection against isoproterenol (ISO)-induced cardiac toxicity in rats [51]. In the Dictionary of Natural Products, 27 alkaloids were identified including different thiocarbamate and isothiocyanates. Furthermore, these thiocarbamate alkaloids are present only in *M. oleifera* giving it a unique profile.

*N. sativa* has been used as a spice and herbal medicine for many centuries throughout the world, especially in Indian and Middle Eastern traditional medicine systems such as Unani and Ayurveda. *N. sativa* is native to the Mediterranean, Northern Africa, the Middle East, and Western Asia, and seems to be underrepresented in GBIF (Table 5; Figure 2). It is used traditionally for treating asthma, bronchitis, rheumatism, and related inflammatory diseases [52,53]. Today it is cultivated for culinary and medicinal uses, such as treatments for diabetes, liver steatosis, and asthma. Indazole alkaloids, specifically nigellicine are found in trace amounts only in seeds with reported antibacterial and lipid-lowering effects [54]. Intervention studies for dietary supplement predominate in the literature, with other clinical trials including treatments for COVID-19, chronic periodontitis, dyslipidemia, asthma, and major thalassemia [55,56,57,58,59].

Nontherapeutic uses also drive the importance of a species. *Triptergium wilfordii* (known colloquially as “Thunder god vine”) has an essential role as an ‘eco-friendly’ insecticide. *Triptergium wilfordii* is commonly used in Traditional Chinese medicine and has marketing authorization in China for treating rheumatoid arthritis. Anti-inflammatory and immunosuppressive properties have been found in seven different sesquiterpene alkaloids isolated and identified from *Triptergium wilfordii* root bark [60]. Most of the seven alkaloids demonstrated insecticidal activity to four insect species, with wilforine being the main insecticidal chemical constituent [61].

In case of *Carthamus. tinctorius*, serotonin derivatives are found and regarded as primary bioactive compounds. Many such as 4,4″-Bi[N-4-hydroxycinnamoylserotonin]; (*E*,*E*)-form are unique to *C. tinctorius* and cannot be found in other species. This high abundance may also have facilitated the identification of new metabolites such as safflospermidine A and B, N1,N5,N10-(*Z*)-tri-p-coumaroylspermidine, and N1,N5,N10-(*E*)-tri-p-coumaroylspermidine from the flowers of *C. tinctorius* [62].

On the other hand, *Aconitum carmichaeli* Debeaux shows a low biodiversity with merely 336 occurrences. It is a ‘classical’ alkaloid containing species with over 100 alkaloids identified (Table 5) and typically contains C19-diterpenoid [63] alkaloids which are structurally complex and difficult to extract. Unprocessed Fuzi contains a high concentration of diester-diterpenoid alkaloids such as aconitine and mesaconitine, causing curariform toxicity and aconitine-type toxicity [64]. However, numerous detoxification methods, including hydrolysis, Paozhi processing, decoction, and combination with other botanical drugs, are claimed to significantly reduce its toxicity profile. Although these approaches are acceptable to Chinese regulatory authorities, they are not generally not approved by other regulators [65] such as the FDA or MHRA. On the other hand, among the top eight species alkaloids in *A. carmichaelii* have been included in specific programmes of drug discovery. For example, *A. carmichaelii* is used in the treatment of cardiovascular disease [66] as a traditional Chinese medicine. The bioactive diterpenoid alkaloids demonstrate effective in vitro suppression in cancer cell lines [67]. However, its development is controversial in terms of risk benefits assessments, with the current research and development being limited in essence to China [68]. Similarly, in the case of *T. wilfordii,* the associated toxicity and formulation issues prevent the widespread usage in clinical settings. These two cases demonstrate biodiversity related considerations are part of a more complex amalgamation of feasibility criteria.

*Mitragyna speciosa* (Kratom) is known to contain psychoactive mitragynine, mainly found in the leaves. In recent years it has become increasingly popular as a recreational drug, and the GBIF demonstrated a low level of distribution and a limited increase in biodiversity data, mostly limited to southeast Asia (Figure 2). Since the extracts contain over 60% of the main bioactive mitragynine [69], it is strictly regulated for both laboratory access and cultivation. Furthermore, many Southeast Asian countries such as Thailand have banned the use of Kratom.

*Ophiopogon japonicus* (Maidong), contains the alkaloid octopamine. Although octopamine is a mild psychoactive agent banned by the World Anti-Doping Agency (WADA) for competitive sports, it is still accessible to the general public. Despite limited evidence for the use of octopamine improving athletic performance [70], it is possess wide appeal in the form of octopamine based supplements marketed to enhance mental and physical capabilities. Amounts of alkaloid found in *O. japonicus* are not known, but Maidong is listed as an edible Chinese medicine by the Chinese Ministry of Public Health due to its established safe use [71]. Therefore, registering such herbal supplements under an FDA scheme is possible and does not normally require additional preclinical or clinical trials to be on the market. This species is cultivated both for ornamental and medical uses, and its research and development certainly benefit from its ease of access (Figure 2).

## 3. Conclusions

Natural products are underrepresented in drug discoveries due to misinterpreting some empirical rules, as well as computing designs that filter out the ‘hit’ molecule that has desired properties for therapeutic uses. Basing strategies exclusively on such rules and filters will discriminate against alkaloids and other natural products with potential therapeutic properties. There is, however, growing interest in developing natural products (especially alkaloids) into potential therapeutic agents. Six years after the initial analysis by Amirkia and Heinrich (2016), a re-assessment of the evidence provides a much stronger empirical basis, especially as it relates to a species’ geographical abundance. This analysis demonstrates the usefulness of assessing a species’ biodiversity in the context of its current medical use. The GBIF is an extremely useful tool for this, even though there remain inconsistencies in the data, and some groups such as cultivated plants are underrepresented. This analysis not only incorporates species yielding compounds for use as licensed medicines, but also alkaloid containing species used as functional foods, herbal medicines or botanicals.

This analysis also provides empirical evidence for the complexity of analysing ‘big data’, and in this case specifically big data in the context of biodiversity and medicine development. The causes underlying the variations observed are often difficult to discern

Here, we demonstrate quantitatively that there is an interrelationship between the use of a species as a medical agent and pushes for agricultural products to become multifunctional commodities. These alkaloids are often associated with uses in traditional medicine due to extensive cultivation, quality control, and established use. The focus here is on species which have seen wide medicinal use (however, since these are also commonly use for culinary purposes, this study does not address the risks of overexploitation due to factors such as intensive harvesting). There are of course numerous examples of species which are overexploited, which generally seems to be a concern with trade items that are either less well controlled from a regulatory perspective, or part of some illicit trade. Here, we highlight both the likelihood of a natural product being developed into a medicine (with a more extensive GBIF dataset compared to 2015) and the push for a more agro-economical production of the *materia prima.* Therefore, parallel to the previous arguments by Amirkia and Heinrich (2014) [18], here we look at the interrelationships between drug development and biodiversity (i.e., that the success of a development process will also require an agroeconomic development).

We also incorporate examples of widely distributed alkaloids and compare these with a set of uncommon alkaloids from rare species. The high number of simple alkaloids and the unique chemical properties of alkaloids synthesized by plants enhances accessibility for research. This offers parameters to modify molecules due to its lower molecular size, weight, and hydrogen donating/accepting capability. Less extraction and purification steps are needed for isolating the bioactive compounds. Simple alkaloids without a high biodiversity profile did not exhibit research interest with the exception of species native to South East Asia.

For the success of a product on a global scale, an integrated and sustainable strategy is needed which ascertains a long-term supply of the *materia prima*. The examples of species at risk due to overexploitation also result in a collapse or dramatic decline of a product. Therefore, the economic success of such high-value products depends on sustainable sourcing and production. The production of the *materia prima* currently faces ever-increasing challenges, including climatic instability, overexploitation, and poor management of the value chains [72].

## 4. Materials/Methods

### 4.1. GBIF Analysis

The initial data set of 27,683 alkaloids was imported from the Dictionary of Natural Products web portal (dnp.chemnetbase.com/), last accessed on 31 August 2020, into Microsoft Excel 2010. GBIF (www.gbif.org/occurrence) data, last accessed on 20 October 2020, was manually queried and exported from the GBIF web portal into Microsoft Excel. The initial dataset for alkaloids and GBIF data was gathered by Heinrich and Amirkia (2014). The dataset in 2014 included the alkaloid compounds, species that contain the particular alkaloid compounds, and their occurrences in 2014. For New GBIF 2020 data gatherings, only organisms that are identified at the species level are included for occurrences data gatherings. Filtration criteria include ambiguous chemical names, unclear nomenclature without species name, or an indicated empty source. In total from initial data sets of 27,683 alkaloids, 3410 alkaloids were filtered out based on these criteria, leaving 24,273 alkaloids for analysis.

The GBIF 2020 (www.gbif.org/species) occurrences data, accessed on 20 October 2020, was gathered using the AI-Powered visual website scraper ScrapeStorm for alkaloid containing species. It was then exported to Microsoft Excel for direct comparison and statistical analysis. For alkaloids demonstrating sources in two or more species, the occurrences for each of the containing species are added up. For example, DNP reports alkaloid Akuammine can be found under the heading of five taxa: *Picralima nitida* (Stapf) T.Durand and H.Durand*, Picralima klaineana* (Stapf) T.Durand and H.Durand*, Cabucala erythrocarpa* (Vatke) Markgr., and *Vinca herbacea* Waldst. and Kit. Occurrences in GBIF for these four plant species total 2001 (758, 8, 79, and 1156, respectively). A scraping software was used to eliminate human error when manually extracting big data. Any abnormalities (such as increases or decreases of several orders of magnitude in the 2014 data compared to the average value) were double-checked manually to ensure data accuracy. GBIF occurrences data that demonstrated increase with no initial occurrences are omitted in the calculation for the percentage increase or decrease.

### 4.2. Search Strategies for Used as Supplements/Botanicals

To assess alkaloids’ significance as bioactive compounds, we used the Core Collection citation indexes using the overarching term “ethnopharmacology” for topic search between 2014 and 2020. These citation indexes include the Arts & Humanities Citation Index (A&HCI), Science Citation Index Expanded (SCI-EXPANDED), Social Sciences Citation Index (SSCI), Conference Proceedings Citation Index—Science (CPCI-S), Conference Proceedings Citation Index—Social Science & Humanities (CPCI-SSH), and Emerging Sources Citation Index (ESCI). In total, 866 records matched the query out of the 21,301,751 total records between 2014 and 2020 on Web of Science. Citation report was generated and extracted to excel for the 866 records. Articles associated with specific species are analysed and summed up, and the total numbers of citations can be calculated from the published articles regarding the species. The top 8 cited alkaloid containing species are used for further analysis. To ensure species contain alkaloid chemical constituents, species are cross-referenced with Natural Products web portal (dnp.chemnetbase.com/) of 27,683 alkaloids, last accessed on 30 January 2020, that had previously been exported to Excel.

Analysis includes the species’ pharmacology, cultivation, research trend, clinical trials, and biodiversity. We excluded research papers that only addressed the genus or did not mention natural sources. Research trends are calculated based on number of articles published per year on Web of Science. Pharmacological significance and laboratory analysis for alkaloids were queried manually using the Chemical abstract service SciFinder (scifinder.cas.org) and PubChem (pubchem.ncbi.nlm.nih.gov) accessed on 25 February 2021. Only references containing the full name of a chemical are queried to avoid any ambiguity. Clinical data for the number of trials that took place for a species were queried manually on clinical.gov, including all the synonyms and scientific names mentioned between 2014 to 2020. The search included all studies such as recruiting, not recruiting, completed, and preliminary studies. Geographical distribution data for each top-cited species using their validated taxonomical nomenclature were searched on both GBIF (www.gbif.org/occrrences/map) databases accessed on 20 October 2020, and Kew Backbone Distribution databases found on http://www.plantsoftheworldonline.org/ that are accessed on 18 February 2021. The species nomenclature was validated taxonomically using http://mpns.kew.org/mpns-portal/ or http://www.plantsoftheworldonline.org/ on 28 February 2021.

## Figures and Tables

**Table 1:** Comparison of GBIF occurrence for species containing alkaloids—2014 and 2020 (Summary table).

	Data 2014	Data 2020	Folds-Increase
**Overall alkaloid: 24,325**			
Average occurrences of a species per alkaloid compound	1295	11,210	8.66
Standard Deviation of occurrences for a species per alkaloid	8703	49,503	8.33
**Medicinal alkaloids only data: 52**			
Average occurrences of a species per alkaloid	17,952	60,991	3.39
Standard Deviation of occurrences for a species per alkaloid compound	35,595	125,243	3.55
**Non-Medicine alkaloids only data: 24,273**			
Average occurrences of a species per alkaloid	1257	11,099	8.83
Standard Deviation of occurrences of a species per alkaloid	8509	72,261	8.5

**Table 2:** Record of alkaloids used in marketed medicines, drugs, and clinical environments. The table was updated table based on Heinrich and Amirkia data from 2014 [18]. Table provides an overview of licensed alkaloids on the market as of 2020. Other sources are determined from DNP and associated uses are queried on PFAF.

Alkaloid	Therapeutic Indications	Source; Other Uses of the Source
Aconitine	Rheumatism, neuralgia, sciatica	*Aconitum napellus* L. and others; reduce fever, pneumonia, laryngitis
	Antiviral agent, pharmaceutical aid used to extend shelf-life of whole blood	Widespread throughout animal and plant tissues, many uses, especially of its derivatives as antiviral agents
Ajmaline	Antiarrhythmic agent	*Rauvolfia serpentina* (L.) Benth. ex Kurz and others; hypertension, ‘insanity’, increase uterine contraction
Atropine	Antispasmodic, anti-parkinson, cycloplegic drug	*Atropa bella-donna* L. and others; to treat peptic ulcers, relives intestinal colic, pupil dilation agents
Berberine	Eye irritations, AIDS, hepatitis	*Berberis vulgaris* L. and others; anti-cancer, anti-inflammatory, antioxidant
Boldine	Cholelithiasis, vomiting, constipation	*Lindera aggregata* (Sims) Kosterm., *Peumus boldus* Molina and others; aromatic, decongestant, diuretic
Caffeine	Neonatal apnea, atopic dermatitis	*Theobroma cacao* L. and others; food additive, emollient, angina treatment, hypertension treatment
Canescine	Antihypertensive agent	*R. serpentina* and others; anti-inflammatory agent, uterine contractions agents
Cathine	Anorectic drug	*Catha edulis* (Vahl) Endl. and others; wakefulness, psychostimulatory effects
Cinchonidine	Increases reflexes, epileptiform convulsions	*Cinchona tucujensis* H.Karst. and others; fever treatment, malaria treatment
Cocaine	Local anaesthetic	*Erythroxylum coca* Lam. and others; GI symptoms treatment, altitude sickness treatment
Codeine	Antitussive, analgesic	*Papaver somniferum* L. and others; antioxidant, antimutagenic, and anticarcinogenic effects
Colchicine	Amyloidosis treatment, acute gout	*Colchicum autumnale* L. and others; analgesic, antirheumatic, cathartic and emetic
Diethanolamine	Base used in pharmaceuticals, etc.	For example, in *Schinopsis balansae* Eng.; *used in dermatological products, as a surfactant, not an active pharmaceutical ingredient*
Emetine	Intestinal amoebiasis, expectorant drug	*Alangium lamarckii* Thwaites and others; emetic, anthelmintic, purgative
Ephedrine	Nasal decongestant, bronchodilator	*Ephedra distachya* L. and others; fruit additive, antiviral agent, allergy treatment
Ergometrine	Postpartum/postabortal hemorrhage	*Claviceps urpurea var.* purpurea (Fr.) Tul. and others; migraine treatment, Parkinson’s disease treatment, antitumor agent
Ergotamine	Migraine treatment	*Claviceps purpurea var. purpurea* (Fr.) Tul. and others; migraine, Parkinson’s diseases. Antitumor
Eserine	Ophthalmology, antidote/poisoning	*Physostigma venenosum* Balf. and others; glaucoma, myasthenia gravis treatment
Galanthamine	Muscle relaxant, Alzheimer’s	*Galanthus woronowii* Losinsk. and many other species of the Amarylidaceae; emmenagogue, treatment of traumatic injuries to nervous system
Hydrastine	Gastrointestinal disorders	*Corydalis fimbrillifera* Korsh. and others; treatment for depression, hypertension, intestinal spasms
Hyoscine	Motion sickness	*Datura stramonium* L. and others; analgesic, anthelmintic and anti-inflammatory
Hyoscyamine	Antispasmodic, antiparkinson, cycloplegic drug	*Hyoscyamus niger* L. and others; pain killer, sedation, diuretic
Lobeline	Anti-smoking, asthma, cough	*Lobelia inflata* L. and others; antispasmodic, respiratory stimulant
Morphine	Pain relief, diarrhoea	*Papaver somniferum* L. and others; antispasmodic, expectorant, antitussive
Narceine	Cough suppressant	*Papaver somniferum* L. and others; antispasmodic, expectorant, antitussive
Nicotine	Anti-smoking	*Nicotiana tabacum* L. and others; relaxant, antispasmodic, discutient, diuretic
Noscapine	Cough suppressant	*P. somniferum* and others; antispasmodic, expectorant, antituissive
Omacetaxine mepesuccinate	chronic myeloid leukaemia (CML)	*Cephalotaxus fortune and others; anti-cancer activities*
Papaverine	Vasodilator, gastrointestinal disorders	*P. somniferum.* and others; antispasmodic, expectorant, antitussive
Pelletierine	Tenia infestations	*Punica granatum* L. and others; astringent, anti-bacterial, antiviral
Pilocarpine	Miotic in treatment of glaucoma, leprosy	*Pilocarpus microphyllus Stapf ex Wardlew.* and others; hair tonic, epilepsy, GI disorders treatment
Quinidine	Ventricular and supraventricular arrhythmias, malaria, cramping	*Cinchona officinalis* L. and others; fever, spasm relax, neuralgia
Quinine	Malaria, babesiosis, myotonic disorders	*Cinchona officinalis* L. and others; fever, spasm relax, neuralgia
Raubasine	Vascular disorders	*Catharanthus roseus* (L.) G.Don and others; anticancer, hypoglycaemic agent, emetic
Rescinnamine	Hypertension	*R. serpentina* and others; hypnotic, increase urine contractions, treat wounds and itches
Reserpine	Hypertension, psychoses	*R. serpentina* and others; hypnotic, increase urine contractions, treat wounds and itches
Rotundine	Analgesic, sedative, hypnotic agent	*Stephania epigaea* H.S.Lo and others; reduce fever
Sanguinarine	Antiplaque agent	*Sanguinaria canadensis* L. and others; fever, rheumatism, expectorant
Sparteine	Uterine contractions, cardiac arrhythmias	*Lupinus pusillus* Pursh var. pusillus and others; treatment for haemostatic, ears and eyes disorders
Strychnine	Eye disorders	*Strychnos wallichiana* Steud. ex A.DC. and others; leprosy, antidote for rabies, ulcers treatment, rheumatism treatment
Synephrine	Vasoconstrictor, conjunctival decongestant, weight loss	*Citrus x aurantium* L. and others; food, stimulant, appetite suppressant, trat. nausea
Taxol	Mamma and ovary carcinoma	*Taxus brevifolia* Nutt. and others; treatment for diabetes, cancer treatment
Theobromine	Asthma, diuretic agent	*Theobroma cacao* L. and others; food, emollient, angina treatment, hypertension treatment
Theophylline	Asthma, bronchospasms	*Camellia sinensis* (L.) Kuntze and others; cancer prevention, lower cholesterol, anti-parkinsons
Turbocuranine	Muscle relaxant	*Chondrodendron tomentosum* Ruiz and Pav.; oedema, kidney stones, persistent urinary tract infections
Vinblastine	Hodgkin’s disease, testicular cancer, blood disorders	*Catharanthus roseus* (L.) G.Don and others; anticancer, hypoglycaemic agent, emetic
Vincamine	Vasodilator	*Vinca minor—*L. and others.; arteriosclerosis, dementia, cerebral stimulant
Vincristine	Burkitt’s lymphoma	*Catharanthus roseus* (L.) G.Don and others; anticancer, hypoglycaemic agent, emetic
Vindesine	Chemotherapy	*C. roseus* and others; anticancer, hypoglycaemic agent, emetic
Yohimbine	Aphrodisiac, urinary incontinence	*Corynanthe johimbe* K. Schum. and others; cereberal stimulant, anti-diuretic, local anaesthetic

**Table 3:** Examples of alkaloids and source species with over one million total occurrences in the GBIF. A source species is only included if it is identified botanically with the species level in the DNP and data on the genus level and above are excluded. Therefore, these are examples of simple alkaloids surpassing 1 million total occurrences and do not account for all examples. Occurrences collected at genus level include those for species. Pharmacological study references of alkaloids are queried on the chemical abstract service (CAS).

Alkaloid	Species	Pharmacological Studies Using the Alkaloid	Occurrences of the Species	Total Occurrences (Species and Genus Level)
4-Hydroxy-1,1-dimethylpyrrolidinium-2-carboxylate/achillein	*Achillea millefolium* L.*, Betonica officinalis* L., *Marrubium vulgare* L., etc.	72	1,292,871	2,278,697
2,6-Benzoxazolediol; 6-Me-ether	*Coix lacryma* L., *Triticum aestivum* L., *Zea mays* L., *Secale cereale* L., etc.	12	1,292,114	1,263,403
2,4-Undecadiene-8,10-diynoic acid; (2E,4E)-form, 2-Methylpropylamide	*Chrysanthemum frutescens* L., *Achillea macrophylla* L., *Achillea ptarmica* L., etc.	2	1,032,568	1,360,771
8-Azabicyclo[3.2.1]octane-1,2,3,4-tetrol/calystegine	*Atropa belladonna* L., *Solanum tuberosum* L., *Solanum dulcamara* L., etc.	160	1,018,893	2,129,193
2,4-Undecadiene-8,10-diynoic acid; (2E,4E)-form, 2,3-Didehydropiperidide	*Otanthus maritimus* (L.) Hoffmanns. and Link, *Achillea millefolium* L. and *Achillea ptarmica* L.	8	1,016,004	1,310,187
Homostachydrine; (S)-form	*Medicago sativa* L. and *Achillea millefolium* L.	54	1,003,339	2,303,110

**Table 4:** Simple alkaloids with low chemo-diversity (a low count in GBIF occurrences) and number of reported published research articles on species from Web of Science between 2014 and 2020. ^1^ Majority of the studies on the compounds focuses on chemical synthesis (>50%) ^2^ Used as traditional medicine.

Species	Alkaloid	Occurrences	Species Distribution	Reported Articles on the Species in WoS (2014–2020)	Search for Compounds
*Bellendena montana* R.Br.	Bellendine	1141	New Zealand	1	4
*Bruguiera sexangula* (Lour.) Poir. ^2^	Tropine 1,2-dithiolane-3-carboxylate	832	Southeast Asia	37	2
*Croton tiglium* L. (syn.: Phragmanthera capitata (Spreng.) Balle) ^2^	Crotonine	1002	Southeast Asia	50 (11 under syn.)	1
*Stemona tuberosa Lour.* ^2^	Croomine	614	Southeast Asia	60	40 ^1^
*Mesembryanthemum tortuosum* L. (syn.: *Sceletium tortuosum* (L.) N.E.Br.) ^2^	Mesembrine	288	South Africa	71 (69 under syn.)	163 ^1^
*Schizanthus pinnatus* Ruiz and Pav.	Schizanthine A	690	South America	1	4
*Stapelia hirsuta* L.	*N*-Acetylhordenine	212	South Africa	2	0
*Ulex jussiaei* Webb	Jussiaeiine A	331	Portugal	3	2

**Table 5:** Top cited species in the core collection of the Web of Science that contain alkaloids as bioactive compounds between 2014 and 2020 and have had a wider use in traditional medicine. Excluding well established and common staple food plants like *Zea mays* L., citations are based on Web of Science (WoS) core collections and include the number of times the publication was cited by specific articles from the journals that it covers. *—Very widely cultivated species including as ornamentals usage. ✞—Cultivated to some degree. %—GBIF occurrences indicate a decrease. This is caused by deletion of data by GBIF due to replicates [20].

Source Species	GBIF 2014	GBIF 2020	GBIF Increases	Published Ethnopharma-Cological Papers/Total Papers in WoS Core Collection (2014–2020)	Examples of Alkaloids in the Species	Clinical Trials (n)
*Aconitum carmichaelii* Debeaux *	266	336	70	99/114	C19- and C20-diterpenoid alkaloids	1
*Carthamus tinctorius* L. *	3225	10967	7742	409/749	Tetrahydro-β-carboline	35
*Mitragyna speciosa* Korth. ✞	85	147	62	204/230	Mitragynine	2
*Moringa oleifera* Lam. *	1720	2349	629	2544/2931	*Marumoside A* and *marumoside* B	12
*Nigella sativa* L. *	191	580	389	2206/3073	Nigeglanine	16
*Ophiopogon japonicus* (Thunb.) Ker Gawl. *	3270	4270	1000	149/192	Octopamine and Ancistrobrevine B	1
*Sophora flavescens* Aiton *	2601	2622	21	339/422	Matrine-type, cytisine-type, anagyrine-type, and lupinine-type alkaloids; Sophora flavescent alkaloids (SFAs)	1
*Tripterygium wilfordii Hook. f.* ✞	2359	2713	354	641/694	Wilfordine, wilforine, wilfornine, wilfortrine, ebenifoline E-II and cangorinine E-1	12

## Data Availability

Not applicable.

## Supplementary Materials

The following are available online, Figure S1: Top 25 species listed in GBIF based on biodiversity occurrences. Identified alkaloids are extracted from Dictionary of Natural Products (DNP) and medicinal/food uses are provided by PFAF Plants for a future database 2020 about specific species.
