# Supplementary material for: Alkaloids Used as Medicines: Structural Phytochemistry Meets Biodiversity—An Update and Forward Look
Source: Molecules. 2021 Mar 25;26(7):1836. doi: 10.3390/molecules26071836 (PMC8036335; doi:10.3390/molecules26071836)
Supplement: Supplementary file 1 [file molecules-26-01836-s001.pdf]

The abundance of alkaloids-containing species and its therapeutic investigation

Supplementary information

Supplementary Table 1: Top 25 species listed in GBIF based on biodiversity occurrences. Identified alkaloids are extracted from Dictionary of Natural Products (DNP) and medicinal/food uses are provided by PFAF Plants for a future database 2020 about specific species.

Species	Occurrences	Identified Alkaloids	Selected medicinal and food uses
Triticum aestivum L.	858198	5	Food uses; antipyretic, sedative, antihidrotic, improve female fertility
Trifolium repens L.	815131	2	Treatment of coughs, colds, fevers and leucorrhoea
Achillea millefolium L.	814224	8	Tonic, anti-inflammatory, anti-spasmodic, diaphoretic
Hedera helix L.	603279	1	Antibacterial, emetic, vasodilator
Corylus avellana L.	600108	3	Food use; astringent, diaphoretic, febrifuge, nutritive and odontalgic
Fagus sylvatica L.	586202	2	antacid, antipyretic, antiseptic, antitussive, expectorant, odontalgic
Pinus sylvestris L.	583687	1	antiseptic, diuretic and expectorant
Calystegia sepium (L.) R.Br.	581166	3	demulcent, diuretic, febrifuge, poultice and strongly purgative
Galium aparine L.	522214	3	Diuretic, antiphlogistic, aperient, diaphoretic, diuretic, febrifuge
Daucus carota L.	511657	3	Food; anthelmintic, carminative, deobstruent, diuretic, galactogogue, ophthalmic, stimulant
Lolium perenne L.	510788	7	Diarrhoea, haemorrhages and malaria
Sambucus nigra L.	495020	6	diaphoretic, diuretic, expectorant and haemostatic
Picea abies (L.)H.Karst.	466427	6	antibiotic, antiseptic, balsamic, expectorant, sedative
Equisetum arvense L.	444940	2	anodyne, antihaemorrhagic, antiseptic, astringent, carminative, diaphoretic,

			diuretic, galactogogue, haemostatic and vulnerary
Phalaris arundinacea L.	426049	4	None
Rosmarinus officinalis L.	414732	1	Antiseptic, anti-inflammatory, antioxidant
Hordeum vulgare L.	404700	9	Food use; demulcent, expectorant, galactofuge, lenitive and stomachic
Vaccinium myrtillus L.	399725	2	astringent, diuretic, tonic and an antiseptic for the urinary tract
Pteridium aquilinum L.	397816	1	antiemetic, antiseptic, appetizer and tonic
Rumex obtusifolius L.	392169	1	treatment of jaundice, whooping cough, boils and bleeding
Capsella bursa-pastoris L.	371251	2	antiscorbutic, astringent, diuretic, emmenagogue, haemostatic, hypotensive, oxytocic, stimulant, vasoconstrictor, vasodilator and vulnerary
Oryza sativa L.	368796	17	diuretic, reduces lactation, improves digestion and controls sweating
Vicia sativa L.	362968	15	treat eczema and other skin irritations, and as an antiseptic
Zea mays L.	355260	32	cholagogue, demulcent, diuretic, lithontripic, mildly stimulant and vasodilator
Rubus idaeus L.	346607	1	Food use; anti-inflammatory, astringent, decongestant, ophthalmic, oxytocic and stimulant
